# Virucidal Activity of the Pyridobenzothiazolone Derivative HeE1-17Y against Enveloped RNA Viruses

**DOI:** 10.3390/v14061157

**Published:** 2022-05-27

**Authors:** Rafaela Milan Bonotto, Francesco Bonì, Mario Milani, Antonio Chaves-Sanjuan, Silvia Franze, Francesca Selmin, Tommaso Felicetti, Martino Bolognesi, Soultana Konstantinidou, Monica Poggianella, Chantal L. Márquez, Federica Dattola, Monica Zoppè, Giuseppe Manfroni, Eloise Mastrangelo, Alessandro Marcello

**Affiliations:** 1 Laboratory of Molecular Virology, International Centre for Genetic Engineering and Biotechnology, Padriciano 99, 34149 Trieste, Italy; rafaela.bonotto@icgeb.org (R.M.B.); tatikon11@gmail.com (S.K.); poggiane@icgeb.org (M.P.); chantalarwen@gmail.com (C.L.M.); federica.dattola@icgeb.org (F.D.); 2 Biophysics Institute, CNR-IBF, Via Celoria 26, 20133 Milano, Italy; francesco.boni@ibf.cnr.it (F.B.); mario.milani@unimi.it (M.M.); monica.zoppe@cnr.it (M.Z.); 3 Dipartiment of Biosciences, Università di Milano, Via Celoria 26, 20133 Milano, Italy; antonio.chaves@unimi.it (A.C.-S.); martino.bolognesi@unimi.it (M.B.); 4 Fondazione Romeo e Enrica Invernizzi, University of Milano, Via Celoria 26, 20133 Milano, Italy; 5 Department of Pharmaceutical Sciences, Università di Milano, Via Giuseppe Colombo 71, 20133 Milano, Italy; silvia.franze@unimi.it (S.F.); francesca.selmin@unimi.it (F.S.); 6 Deptartment of Pharmaceutical Sciences, Università degli Studi di Perugia, Via Fabretti 48, 06123 Perugia, Italy; tommaso.felicetti@unipg.it (T.F.); giuseppe.manfroni@unipg.it (G.M.)

**Keywords:** antivirals, enveloped viruses, flavivirus, virucidal effect, reporter replicon particles

## Abstract

Pyridobenzothiazolone derivatives are a promising class of broad-spectrum antivirals. However, the mode of action of these compounds remains poorly understood. The HeE1-17Y derivative has already been shown to be a potent compound against a variety of flaviviruses of global relevance. In this work, the mode of action of HeE1-17Y has been studied for West Nile virus taking advantage of reporter replication particles (RRPs). Viral infectivity was drastically reduced by incubating the compound with the virus before infection, thus suggesting a direct interaction with the viral particles. Indeed, RRPs incubated with the inhibitor appeared to be severely compromised in electron microscopy analysis. HeE1-17Y is active against other enveloped viruses, including SARS-CoV-2, but not against two non-enveloped viruses, suggesting a virucidal mechanism that involves the alteration of the viral membrane.

## 1. Introduction

Emerging and re-emerging RNA viruses are a continuous threat to humanity. Novel and potentially highly pathogenic agents continuously emerge from the large, genetically variable natural pools surrounding us, facilitated by a human-caused reduction in the natural habitats and by the effects of globalization at large [1,2]. However, there is not yet a way to predict which virus may spread next: preparedness to meet such a threat is dependent on the enforcement of sensitive and specific diagnostic and surveillance tools, on platforms for the quick deployment of recombinant vaccines and on the availability of broad-spectrum antiviral drugs as a valid first-line defense against future outbreaks [3,4].

The availability of specific antiviral drugs is still limited, and treatments are accessible mostly for endemic diseases such as HIV, HCV and influenza [5,6].

The recent (re)emergence of Ebola, yellow fever virus (YFV), dengue virus (DENV), zika virus (ZIKV), West Nile virus (WNV), and Chikungunya virus (CHIKV), culminating with the current pandemic of SARS-CoV-2, has caused great concern with the compelling request to develop effective antiviral strategies [7,8]. In the early stages of the pandemic, the only drugs available were certain nucleoside analogues, such as ribavirin and remdesivir, originally developed as broad-spectrum antiviral agents [9], whose anti-viral activity has not yet been fully elucidated [10,11]. Only immense international effort made specific inhibitors available against SARS-CoV-2 in less than two years [12,13,14].

Hence, it will be highly advisable to characterize a number of broad-range antiviral drugs as a first-line defence strategy [3].

The majority of pathogenic RNA viruses is characterized by a lipid bilayer which contains specific proteins that bind receptors of the host cells. After attachment, viruses can get internalized by endocytosis and follow a pH-dependent fusion route or can enter the cell directly by fusion of the viral and cellular membranes [15,16,17]. Compounds that interfere with any of these steps could be promising broad-spectrum drug candidates able to block the infection at an early stage [18].

Recently, a new class of pyridobenzothiazolones (PBTZs) showing a broad-spectrum activity against flaviviruses was discovered [19,20,21]. Among the active PBTZs, compound HeE1-17Y (17Y) was one of the most interesting lead compounds, showing antiviral activity on a large panel of flaviviruses including DENV serotypes 1, 2, 3 and 4, ZIKV, different strains of YFV, Japanese encephalitis (JEV), Usutu (USUV) and tick-borne encephalitis (TBEV) viruses [22,23].

In a recent paper, Dejmek et al. [24] showed that PBTZ derivatives significantly inhibit SARS-CoV-2 RNA-dependent RNA polymerase, exerting antiviral activity in cell cultures. However, we believe that the main antiviral effect of PBTZs is not at the level of viral RNA synthesis involving the non-structural proteins (such as RdRp), nor of virion release, as initially thought from biochemical evidence, but it is based on the reduction in virus infectivity [20].

In this work, with the aim of clarifying the mechanism of action (MoA) of 17Y, we performed time course and electron microscopy experiments using WNV reporter replicon particles (RRPs [25]), showing a virucidal activity of the compound. Furthermore, we revealed a selective inhibition of enveloped viruses, suggesting a possible involvement of the viral membrane in the MoA of 17Y.

All the data presented here show that 17Y may be of particular interest to prevent infections from different enveloped viruses, including flavivirus and coronavirus.

## 2. Materials and Methods

### 2.1. Chemistry

HeE1-17Y belongs to the same synthetic batch used for the experiments described in Cannalire et al. [22]. The homologous compound HeE15-2Y has been prepared adapting the synthetic procedure already reported [22], as described in Supplementary Materials and depicted in Scheme S1. HeE1-17Y and HeE15-2Y were solubilized in DMSO at a final concentration of 10 mM and stocked at −20 °C.

### 2.2. Cells and Viruses

Vero E6 cells (ATCC-1586) and HEK 293T (ATCC CRL-3216) were cultured at 37 °C with 5% CO_2_, in Dulbecco’s modified Eagle’s medium (DMEM, ThermoFisher, Paisley, UK) supplemented with 10% fetal bovine serum (FBS, ThermoFisher, Paisley, UK) and antibiotics. CHIKV Asia, WNV EG101, vesicular stomatitis (VSV) Indiana, and SARS-CoV-2 ICGEB-FVG_5 [26] were used for in vitro experiment. Working stocks were propagated and quantified in Vero E6 cells as described previously [27,28]. Preparation of Adeno Associated Virus type 2 Green Fluorescent Protein (AAV2-GFP) and Enhanced Green Fluorescent Protein-Adenovirus 49 (EGFP-AD49) were kindly provided by Lorena Zentilin and Serena Zacchigna (ICGEB, Trieste, Italy), respectively [29]. AAV2-GFP and AD49-GFP were manipulated under biosafety level 2 facility (BSL2). CHIKV Asia, WNV EG101, VSV Indiana and SARS-CoV-2 ICGEB-FVG_5 were manipulated under biosafety level 3 facility (BSL3) according to ICGEB regulations approved by the Safety Committee of the Centre.

### 2.3. RRP Production and Purification

WNV replicon particles (RRPs) carrying a green fluorescent protein (GFP) reporter were produced according to previous work [25,30]. The Pierson’s laboratory generously provided the necessary reagents: WNV CprME, encoding for the structural proteins of WNV strain NY99 (Capsid, prM, E), and WNV Rep, a replicon encoding for the non-structural proteins of WNV strain II and for a GFP reporter gene. HEK293T cells were transfected using polyethylenimine (PEI) with a 6:1 PEI:DNA ratio. Twenty-four hours post transfection, the medium was removed, cells were gently washed with 5 mL of PBS and supplemented with 10 mL of fresh serum-free medium and then they were incubated for 48 h. Seventy-two hours post transfection, supernatant containing the recombinant WNV-RRPs were pooled and centrifuged at 2000 rpm for 10 min at 4 °C and passed through a 0.22 µM filter. Supernatants were added on a 1.5 mL of 20% sucrose cushion into a 14 × 89 mm ultracentrifuge tube and centrifuged at 36,000 rpm for 4 h at 4 °C using a SW-41TI rotor. After gently removing the supernatant and the sucrose cushion with a vacuum pump, the pellet containing the RRPs at the bottom of each tube was resuspended and pooled in 500 µL of NTE buffer (12 mM Tris-HCl pH 7.0, 120 mM NaCl, 1 mM EDTA).

Purified RRP concentration was estimated by comparing the relative intensity of the band of the Envelop (E) protein with that of serial dilutions of BSA in SDS-PAGE. Briefly, from the intensity of E band we extrapolated its amount (~0.25 μgr) and, considering 180 copies of E in every virion and assuming a molecular weight of ~22 MDa, we estimated a concentration of RRPs ~0.1 mg/mL.

The infectivity of the purified WNV-RRPs was assessed by measuring the fluorescence of GFP by flow cytometry (BD Accuri C6) after transduction of Vero E6 cells (40,000 cells/well) using serial dilutions of WNV-RRPs. The percentage of fluorescent cells expressing the GFP 48 h post transduction was converted into the number of infected cells to infer that the purified RRPs were able to mimic the viral infection with a viral titer of 5.25 × 10^7^ RRP/mL.

### 2.4. In Vitro Virucidal and Viability Assays

WNV RRPs were pre-incubated for 1 h with different concentrations of 17Y before transducing Vero E6 cells. After 24 h, cells were harvested and resuspend in PBS to quantify GFP expression by flow cytometry.

Similarly, infectious viruses were pre-incubated with the drug in DMEM at 37 °C for 1 h. DMSO (1%) was used as a vehicle control. After pre-incubation, the preparation was diluted 1:10 to obtain 30 PFU/well (maximum virus concentration for plaque forming quantification) and used to infect a monolayer of Vero E6 cells. Following incubation at 37 °C for 1 h, the virus inoculum was removed, and the plate was washed once with PBS. The infected cells were covered with 800 μL of medium containing 1.5% carboxymethylcellulose (CMC) with DMEM + 2% FBS. Cells were then incubated at 37 °C for 3 days. Finally, cells were fixed with 3.7% PFA and stained with crystal violet. Plaques were counted and values were normalized to controls. The half maximal effective concentration (EC_50_) was calculated using GraphPad Prism Version 7.

AAV2-GFP and EGFP-AD49 were diluted with the drugs as described above and were used to infect HEK293T cells. Medium of cells infected with AAV2 was replaced after 24 h with fresh DMEM medium. Cells were harvested after 48 h from infection for AAV2, or 24 h for AD49, and analyzed for GFP expression by flow-cytometry.

The virucidal activity was calculated from percentage of inhibition, based on the ratio of the number of plaques in each sample to that of the negative control (DMSO).

The cytotoxicity assay was conducted with AlamarBlue (Invitrogen, Waltham, MA, USA) as recommended by the manufacturer’s protocol. Vero cells were seeded at 1 × 10^4^ cells per well in a 96 well plate and incubated at 37 °C overnight. Then, 50 μL of compound at the indicate concentrations were added to 150 μL of medium (final 200 μL). Plates were incubated at 37 °C for 3 days and then the colorimetric reagent was added (20 μL for 4 h). Measurements from 17Y-treated cells were normalized against those from untreated cells. The half maximum cytotoxic concentration (CC_50_) was calculated using GraphPad Prism Version 7.

### 2.5. Time Addition Study of 17Y

To assess the effect of 17Y at different stage of WNV RRP transduction in Vero E6 cells, the compound was added in different conditions. For the early stage, the 17Y was pre-incubated with inoculum at 37 °C or at 4 °C for 1 h, or administrated directly with the inoculum into the cells. For late-stage, cells were treated with the inoculum and after 1, 3 and 5 h the compound was added. After 24 h, all cells were collected and resuspend in PBS to quantify GFP expression by flow cytometry.

### 2.6. WNV RRP Binding Assay

To evaluate attachment, WNV RRPs were pre-incubated for 1 h with 10 μM 17Y and added to the Vero E6 monolayer for 90 min at 4 °C. After several washing steps with ice-cold PBS to remove unbound particles, viral RNA was isolated from cell lysates and quantified by RT-qPCR. Primers for WNV were based on sequences at the 5′ noncoding region of West Nile virus genomic RNA (WNV NCR) FW 5′-CAGACCACGCTACGGCG and WNV NCR RV 5′ CTAGGGCCGCGTGGG (Eurofins Genomics, Ebersberg, Germany). The cellular housekeep gene GAPDH served as a control for intracellular RNA.

### 2.7. Negative Staining Sample Preparation and Image Acquisition

RRPs (viral titer 5.25 × 10^7^ RRP/mL) were treated with 17Y 10 µM (or DMSO 0.1%) for 30 min. Immediately after incubation, 4 μL of sample at a final concentration of ~4.7 × 10^4^ particles/μL was applied onto a glow discharged for 30 s. at 30 mA (GloQube system; Quorum Technologies, Lewes, UK) 400-mesh copper carbon-coated grid (Agar Scientific, Stansted, UK). After 60 s incubation, the excess of sample on grid was removed by gentle side-blotting and the grid was stained with 2% (*w/v*) uranyl acetate solution. Observations were carried out with a Talos L120C (Thermo Scientific, Waltham, MA, USA) with an accelerating voltage of 120 kV. Images were acquired by a Ceta camera 4 k × 4 k with an applied defocus value of −1.5 μM and at a nominal magnification of 22,000×, corresponding to a pixel size of 6.41 Å/pixel at the specimen level.

### 2.8. TEM Images Processing

158 micrographs of untreated sample were imported into RELION-3.1 [31] and CTF corrected (ctffind-4.1.14) [32]. After manual picking (199 particles) and 2D classification (6 classes) automatic picking resulted in the selection of 4128 particles. After the elimination of wrong selections with multiple cycles of 2D classifications, the resulting 3079 particles were reclassified in seven 2D classes, of which 2 were eliminated as the particles presented an elongated/non spherical shape (764 particles). The remaining 2315 particles were grouped in five 2D classes, with 3 different dimensions.

A similar approach was used to analyze the sample treated with 10 µM 17Y. From 190 micrographs, 474 particles were picked and classified into six 2D classes. After the elimination of the class containing elongated/non spherical particles (37 particles), the remaining 437 particles were grouped in five 2D classes with 3 different dimensions.

### 2.9. Preparation of Model Membranes

Two different types of model membrane were prepared by the conventional “Thin Film Hydration Method” using 1,2-dioleoyl-sn-glycero-3-phosphocholine (DOPC, Lipoid, Steinhausen, Switzerland), 1,2-dioleoyl-sn-glycero-3-phosphoethanolamine (DOPE, Lipoid, Steinhausen, Switzerland), sphingomyelin (SM, Sigma Aldrich, Italy) and cholesterol (CHOL, Sigma Aldrich, Italy). Lipids (DOPC and DOPE in the molar ratio 60:40 and SM:CHOL in the molar ratio 70:30) were dissolved in chloroform and transferred in a round flask. The organic solvent was evaporated under reduced pressure (80 mbar), at 50 °C and 80 rpm for 1 h using a rotatory evaporator (RII, Buchi, Italy). The lipid film was re-hydrated for 1 h with a solution containing 5 mM HEPES at pH 7.4 and 150 mM NaCl, to reach the final lipid concentration of 1 mM. Afterwards, both samples were extruded (Avanti^®^ Mini-Extruder, Avanti Polar Lipids, Inc., Birmingham, AL, USA) 5 times through 0.2 μM and 6 times through 0.1 μM polycarbonate membranes to obtain unilamellar vesicles. Particle size distribution and concentration of model membranes were assessed immediately after preparation by Nanoparticle Tracking Analysis (NTA) using a Nanosight NS300 (Malvern Instrument, Malvern, Worcestershire, UK) after 1:1000 dilution in HEPES buffer. All measurements were carried out at 25 °C in triplicate for each sample.

To evaluate the effect of the compound on the membrane stability, each type of model membranes was incubated with 17Y (200 µM) for 7 or 24 h in a static condition at room temperature. Pure DMSO (2%) was used as a negative control. At predetermined times, an aliquot was analyzed by NTA, as previously described, and the data were comparted to those obtained using untreated samples.

### 2.10. Statistical Analysis

Data were analyzed and plotted using GraphPad Prism (GraphPad Software, San Diego, CA, USA). Results are presented as means ± standard deviations. The *p* value was calculated by comparison between percentage of inhibition of infected-treated samples and that of control infected not-treated samples or vehicle. Paired sample *t*-test and one-way ANOVA test were used to compare groups. Significance was reported for *p*-value <  0.05 (*), <0.01 (**) and <0.001 (***).

## 3. Results

### 3.1. HeE1-17Y Inhibits WNV RRP Transduction in a Concentration-Dependent Manner

**Figure 1:**
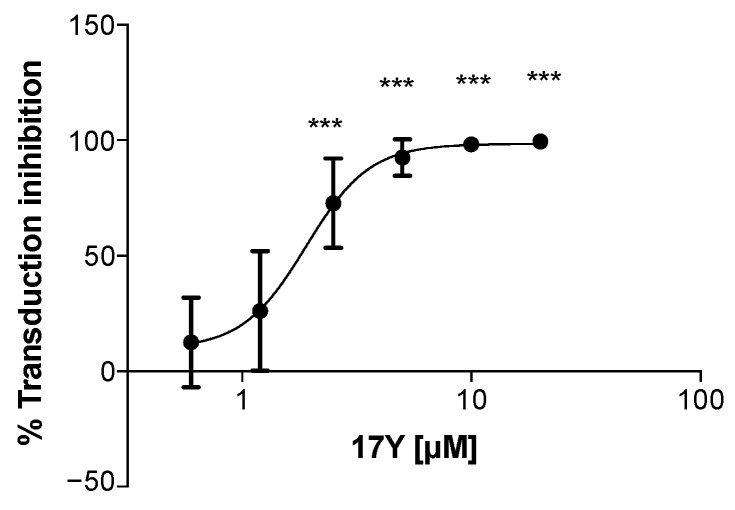
**17Y dose-dependent reduction in WNV RRP transduction efficiency.** WNV RRPs were pre-incubated for 1 h with serial dilutions of 17Y. The mixture 17Y+RRPs was then used to transduce Vero E6 cells for 24 h. The GFP signal (normalized to the signal in the absence of the compound) was used to determine the transduction efficiency. The percentage of transduction inhibition (black dots) was calculated relative to the average of negative control (Vehicle, DMSO) to obtain a dose–response sigmoid curve. The effective concentration resulting in 50% of WNV RRP transduction inhibition (EC_50_) was calculated to be 2.0 ± 0.4 μM. The reported bars represent the mean ± SD from three independent experiments. Significant *p*-values are indicated by *** *p* < 0.001 measured with the one-way ANOVA test, comparing the average percentage of sample transduction inhibition with the negative control (treated with 1% DMSO).

WNV replicon particles (RRPs) carrying a green fluorescent protein (GFP) reporter were used to investigate the mechanism of 17Y antiviral activity. Vero E6 cells were incubated for one hour with sub-saturating dilutions of RRPs in the presence of 17Y. Transduction efficiency was assessed by flow cytometry after 24 h. As shown in Figure 1, incubation with 17Y inhibits WNV-mediated transduction in a concentration-dependent manner with an EC_50_ of 2.0 ± 0.4 μM.

### 3.2. Early HeE1-17Y Administrartion Inhibits WNV RRP Transduction

**Figure 2:**
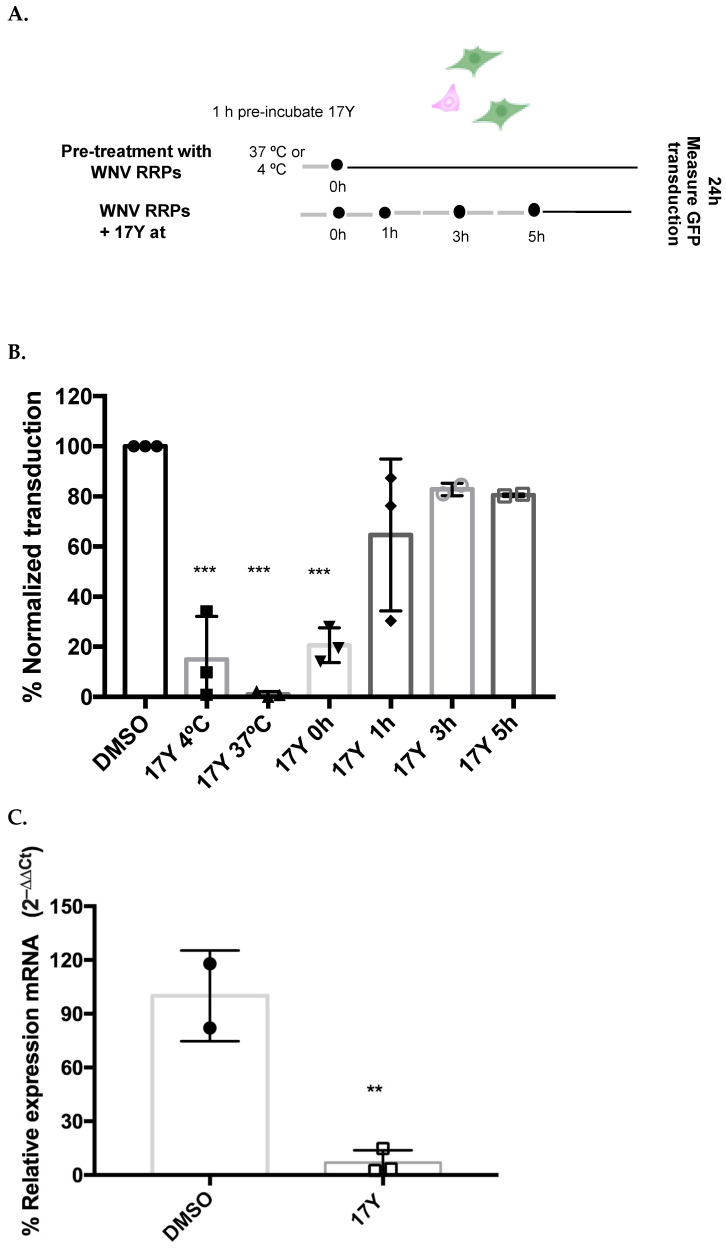
**17Y inhibits WNV RRPs’ transduction acting before attachment.** (**A**) Scheme of the experimental design to study the effect of 17Y at different stages of RRP transduction. (**B**) RRPs were pre-incubated for 1 h with 17Y (10 μM) at 4 °C (Lane 2) or 37 °C (Lane 3) before transduction. Alternatively, the compound was supplemented in the medium after 0, 1, 3 or 5 h following transduction (Lanes 4–5-6–7). Negative control was treated with vehicle DMSO (Lane 1). (**C**) RRPs were pre-incubated with 17Y (10 μM) or DMSO for 1 h before administration to the cells at 4 °C for 90 min. Attached viral particles were evaluated by RT-qPCR (WNV sequences) using the housekeeping gene (GAPDH) as reference. Viral genomes are expressed as percentage of relative expression (double delta Ct method) over negative control (no-transduction -vehicle, DMSO). Significant *p*-values are indicated by *** *p* < 0.001 measured with the one-way ANOVA test, and ** *p* < 0.01 measured with a paired two-tailed *t*-test. The reported bars represent the mean ± SD from three independent experiments (the result of each measure is reported using circle, square or triangle symbols).

To test whether 17Y could affect later steps of infection, cells were transduced with WNV RRPs for 1, 3 or 5 h before replacing the medium and adding 10 μM 17Y (Figure 2A,B, lanes 5–7). The 17Y concentration of 10 μM was chosen because it was the minimum concentration that achieved >95% transduction inhibition of WNV RRPs. As a positive control, 17Y was administered together with the viral particles using two different procedures: 1. pre-incubation of inhibitor and RRPs for 1 h at 37 °C (Figure 2B, lane 3); 2. no preincubation (Figure 2B, lane 4). Viral transduction was inhibited to a lesser extent (~80%, Figure 2B, lane 4) when the compound was added with the RRPs, compared to the pre-incubated sample (inhibition ~99%, Figure 2B, lane 3).

Since flaviviruses undergo conformational changes regulated by temperature [33], we decided to assess whether pre-incubation of 17Y with RRPs for 1 h at 4 °C is still able to inhibit viral infection. In this case, transduction was inhibited but to a lesser extent (Figure 2B, lane 2), showing the role of temperature in 17Y RRP interaction. Conversely, inhibitory activity was completely lost when 17Y was added after infection (Figure 2B, lanes 5–7). Overall, these results indicate that 17Y exhibits antiviral activity only when exposed to the viral particles before transduction.

To study the effect of 17Y on cell attachment [34], WNV RRPs, pre-incubated for 1 h at 37 °C with 10 μM of the compound, were administered to a cell monolayer at 4 °C for 90 min. After several washing steps with ice-cold PBS to remove unbound particles, viral RNA was quantified by RT-qPCR, revealing that 17Y treatment strongly decreases WNV RRP attachment to the cell surface (Figure 2C).

The data obtained, reported in Figure 2, indicate that 17Y acts directly on the virion affecting its ability to attach on the cell membrane, the very first step of viral infection.

### 3.3. Activity of HeE1-17Y on RRPs

Transmission electron microscopy was used to investigate the direct effect of 17Y on WNV RRPs. These particles can be used safely because of their inability to produce infectious viruses [25].

**Figure 3:**
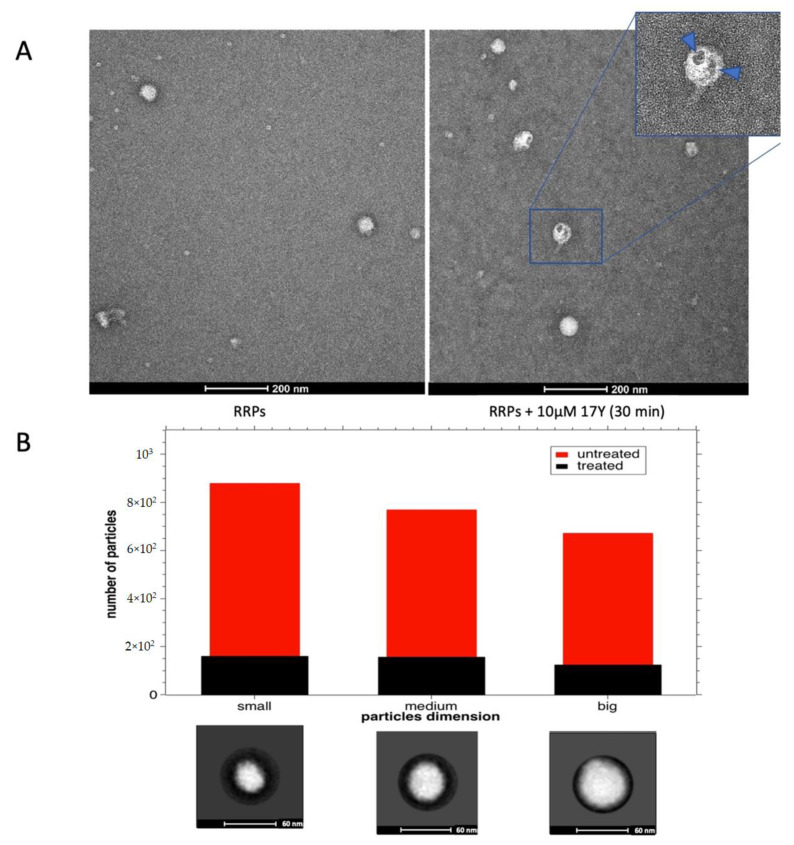
**Negative staining analysis of WNV RRPs treated/not treated with 17Y.** (**A**) Negative staining of WNV RRPs (left) or treated with 10 µM 17Y for 30 min (right). Close-up view of a particle with holes indicated by blue arrows. (**B**) Number of RRP particles not exposed (red bars) or exposed to 17Y (black bars) from 158 and 190 micrographs, respectively. The particles were selected using 2D classification and representative 2D classes are reported below to show each dimension (black circle diameter = 60 nm): small (D~32 nm), medium (D~46 nm) and big (D~56 nm), corresponding to empty, mature, and immature virions, respectively. The error bars represent an assumed error (20%) for the number of particles due to the selection procedure.

Purified RRPs (~0.1 mg/mL) were treated for 30 min at room temperature with/without 10 µM 17Y, applied on 400-mesh copper carbon-coated grids, stained with 2% (*w/v*) uranyl acetate solution, and analyzed with a Talos L120C. Both the sample untreated/treated with 17Y were quite heterogeneous, showing particles with different diameters (Figure 3A). An analysis of 158/190 images was conducted with the RELION-3.1 [31] program after filtering for non-spherical particles and weird shapes. In the untreated samples, the 2315 particles were grouped in five 2D classes, with 3 different dimensions: small (diameter (D)~32 nm; 1 class, 866 particles), medium (D~46 nm; 2 classes, 768 particles) and big (D~56 nm; 2 classes, 670 particles) that should correspond to empty (subviral particles), mature and immature virions, respectively (Figure 3B, red bars [35]). The 17Y-treated samples contained a reduced number of RRPs, some of them with a damaged surface (Figure 3A, right panel), suggesting that the compound induces the disruption of the virions. Indeed, the remaining 437 particles were grouped in five 2D classes with the 3 different dimensions already described: small (1 class, 159 particles), medium (2 classes, 156 particles) and big (2 classes, 122 particles). The treatment with 17Y induces a dramatic drop in the number of particles in each class (Figure 3B, black bars), demonstrating a direct effect of 17Y on virion integrity.

### 3.4. HeE1-17Y Activity against Different Enveloped RNA Viruses

To evaluate whether 17Y is active against different enveloped RNA viruses, we analyzed: (1) WNV, as representative of flaviviruses in the *Flaviviridae* family; (2) CHIKV, an Alphavirus of the *Togaviridae* family; (3) VSV, a *Vesiculovirus* of the *Rhabdoviridae* family; and (4) SARS-CoV-2, a beta-Coronavirus of the *Coronaviridae* family. All the viruses were incubated with a drug concentration range of 0.3–10 μM for 1 h at 37 °C and then used to infect a monolayer of Vero E6 cells. After 1 h, the medium was replaced, and the extent of infection was measured by plaque reduction assay. At the used concentrations, 17Y is not cytotoxic in Vero E6 cells (CC50 > 100 μM) and other cell lines as shown previously [22,23].

**Figure 4:**
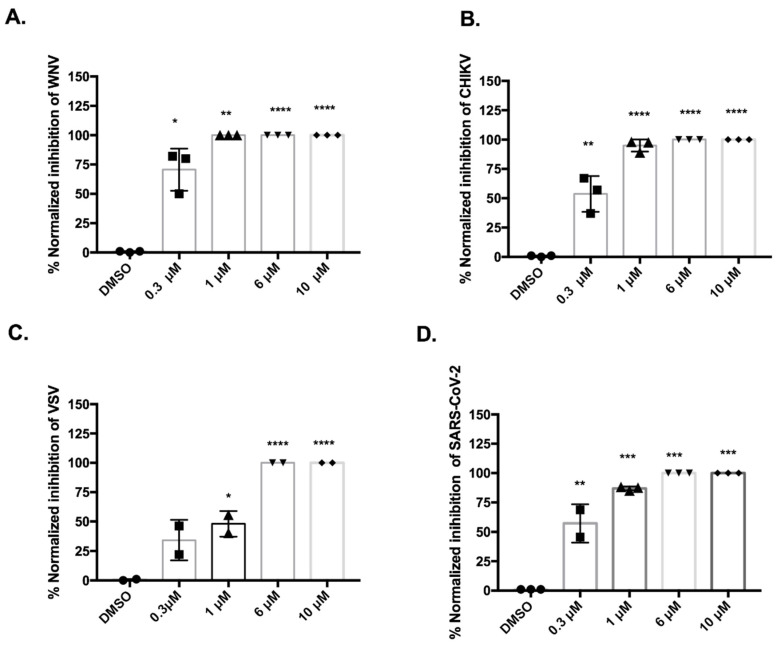
**Virucidal effect of 17Y against enveloped RNA viruses.** Virus inoculum was pre-incubated for one hour with the indicated concentrations of 17Y, or 1% DMSO as vehicle. The inoculum was diluted 1/10 before infection of Vero E6 cells. Plaques were counted 72 h post infection. Inhibition (%) for (**A**) WNV, (**B**) CHIKV, (**C**) VSV, (**D**) SARS-CoV-2 was calculated from the normalised ratio of 17Y-treated over vehicle-treated samples. Columns and bars represent the average and STDV from replicates of two/three independent experiments (the result of each measure is reported using circle, square or triangle symbols). Significant *p*-values are indicated as follows: **** *p* < 0.0001 highly significant; *** *p* < 0.001, ** *p* < 0.01, * *p* < 0.05 weakly significant, measured with a paired two-tailed *t*-test.

17Y strongly inhibited both WNV (Figure 4A) and CHIKV (Figure 4B) infection in a dose-dependent manner with comparable efficiency.

VSV showed 50% of inhibition when pre-treated with 17Y 1 µM, appearing slightly less affected by the drug (Figure 4C) compared to WNV and CHIKV. For SARS-CoV-2, 100% inhibition was obtained at 6 μM 17Y, while activity decreased to 80% and 65% inhibition at 1 and 0.3 μM, respectively (Figure 4D). As a negative control we tested the homologous compound HeE15-2Y, with the same PBTZ scaffold and a *N*-methyl-piperidine instead of the cyclohexyl (Supplementary Material Figure S1), which was inactive against different flaviviruses (unpublished observations). HeE15-2Y did not show any significant inhibitory activity at the maximum concentration tested. These results suggest that 17Y has a specific and broad antiviral activity directed to different enveloped viruses.

### 3.5. HeE1-17Y Is Not Active against Two Non-Enveloped Viruses

**Figure 5:**
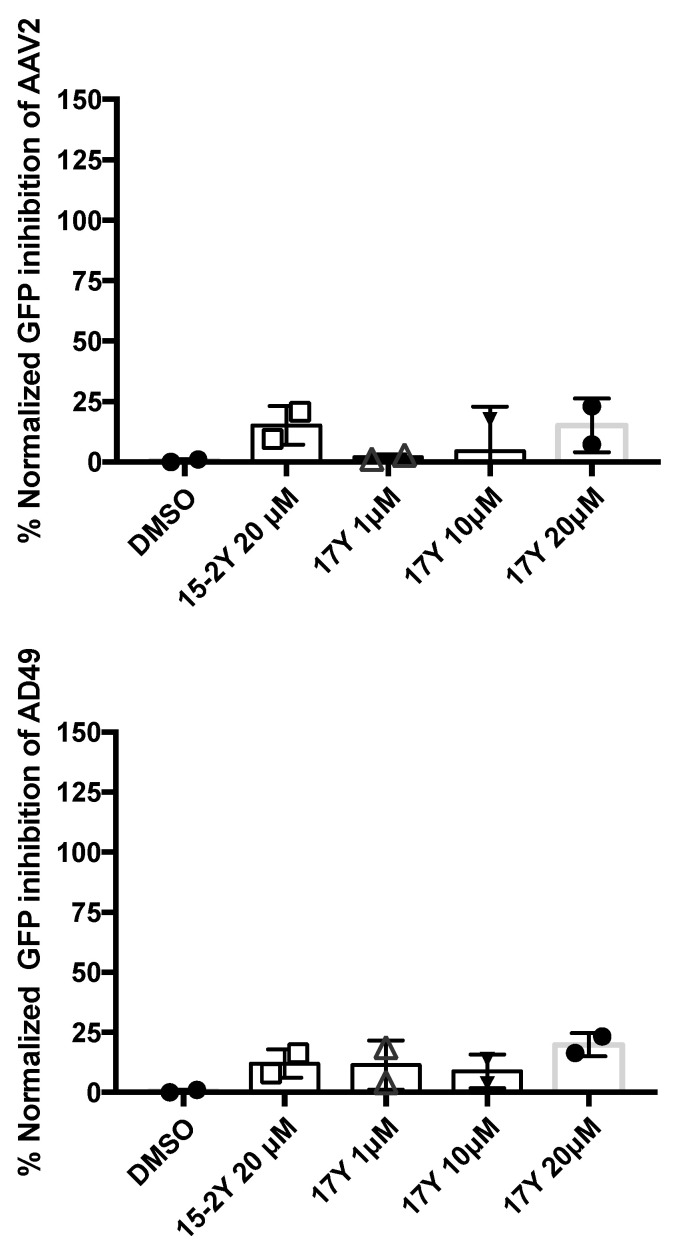
**Lack of virucidal effect of 17Y against non-enveloped viruses:** AAV2 and AD49 were pre-treated for 1 h with 17Y or negative control HeE15-2Y at the indicated concentrations. HEK293T cells were then infected for 24 h for AD49 and 48 h for AAV2. Inhibition (%) was calculated as GFP signal normalized over vehicle (DMSO). Columns and bars represent the average and STDV from replicates (the result of each measure is reported using circle, square or triangle symbols).

To check whether 17Y targets specifically enveloped viruses, we tested the compound against non-enveloped viruses such as the AAV2 vector and AD49, both carrying a GFP reporter [29,36]. Transduction efficiency was quantified measuring intracellular GFP signal as described previously. The AAV2 and AD49 were diluted at an MOI of 1 × 10^4^ and pre-incubated with 17Y for one hour before infecting HEK293T for 24 h (AD49) or 48 h (AAV2). As shown in Figure 5, 17Y and HeE15-2Y were comparable and not significantly active against both AAV2 and AD49.

### 3.6. HeE1-17Y Effect on Selected Liposomes

**Figure 6:**
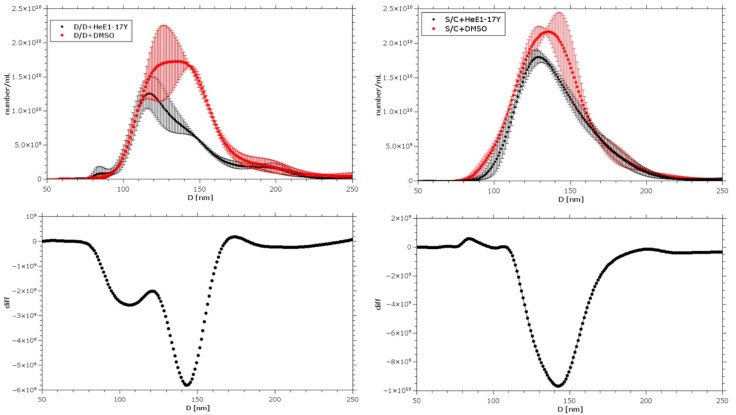
**Liposomes are affected by HeE1-17Y.** Comparison between the liposome diameter distribution after the addition of 17Y (200 μM) or 2% DMSO (vehicle). Left and right D/D and S/C liposomes: distribution averaged on 3 experiments, with standard deviation. Lower panels: differences between the upper curves.

The 17Y activity against enveloped viruses and the RRP disruption observed in EM experiments suggested a possible mechanism of action related to the alteration on the lipidic component of virions. In order to test such a hypothesis, we prepared two different kinds of liposomes as rough models of viral envelopes: DOPE/DOPC (D/D) and sphingomyelin/cholesterol (S/C). Both model membranes were incubated with a saturating amount of 17Y (200 µM) or control (2% DMSO) at room temperature for 7 h (D/D) or 24 h (S/C) and analyzed with Nanoparticle Tracking Analysis (NTA). This technique, widely used to characterize nanosystems and exosomes, allows one to measure the distribution of particle diameters and their concentration. As is evident in the top panel of Figure 6, both types of membrane were perturbed by 17Y compared to the vehicle control. This effect was clearly evident for the liposome population at ~143 nm since the main peak presented a variation in position (i.e., size) and intensity (i.e., concentration) (Figure 6, lower panels). The perturbation effect was more pronounced in the case of D/D-based membranes, where a further population with a diameter of ~70 nm appeared after treatment with 17Y. This smaller population was not found after treatment with DMSO and might have arisen from partial solubilization of the membranes by 17Y. The different behaviour of the two model membranes is expected since sphingomyelin tends to form a more rigid and resistant membrane if compared with DOPE. Nevertheless, also in the case of the S/C-based membrane, a decrease in the main population of the vesicles after treatment with the compound was evidenced, with respect to the control, along with a shift of the main diameter towards smaller values.

## 4. Discussion

In this work, we characterized the broad-spectrum antiviral activity and mechanism of action of HeE1-17Y against different enveloped viruses, including SARS-CoV-2. The compound was originally designed based on the 1*H*-pyrido[2,1-*b*][1,3]benzothiazol-1-one scaffold (called PBTZ) [22,37]. PBTZ derivatives showed antiviral activity against a number of flaviviruses of human relevance [22]. Recently Dejmek et al. reported HeE1-2Tyr, the progenitor of the PBTZ class, and its derivates as inhibitors of SARS-CoV-2 RdRp with an IC_50_ in the µM range (IC_50_ of 27.6 ± 2.1 µM) [24]. Moreover, the authors showed a good anti-viral activity in cell culture experiments, with an EC_50_ ~ 1 µM, in agreement with our results for 17Y (Figure 4D; EC_50_~0.3 µM). The difference between the IC_50_ value against SARS-CoV-2 RdRp and EC_50_ value in cell cultures suggests that process(es) other than RdRp inhibition may contribute to the MoA of PDBZs. In our previous work [20], we speculated that the antiviral activity of PDBZ compounds was related to the formation of non-infectious virions rather than on its effect on RNA replication, as initially thought [19].

Among the most promising PBTZs, 17Y showed a robust inhibitory activity against flaviviruses in the low-micromolar range. In this work, we show that the pre-incubation of the viral particles with 17Y causes a complete inhibition of infection (Figure 2). Moreover, 17Y is inactive when added post-entry, suggesting that its prevalent antiviral MoA is directed against the virions.

The imaging of virus particles by electron microscopy showed the disappearance of RRPs in treated samples, with damaged residual virions (Figure 3). The virucidal activity of 17Y is not limited to WNV but could be extended towards different enveloped RNA viruses belonging to distant families (CHIKV, VSV, SARS-CoV-2). To note, 17Y was previously shown to be unable to inhibit CHIKV, although the quantification readout was based on RNA level, not on infectious virus [22].

17Y was not active against two non-enveloped viruses, AAV2 and Ad49, suggesting a mechanism of action related to the lipidic component of virions. Accordingly, we demonstrated a direct effect of 17Y on model lipidic membranes and specifically against particles with a dimension of ~140 nm (Figure 6). This observation is suggestive of the dependence of the activity on liposomes geometry and on particular membrane curvature [38]. Irrespective of this, since the direct effect on liposomes is small, we cannot exclude the fact that the MoA might involve the interaction between the envelop and viral proteins.

Several antiviral drugs have been reported to be able to interfere with the lipid content of the virion. For instance, the lipidomimetic compounds IBS70, J391B and J582C were shown to affect the membrane of HIV-1 virions thus impacting entry [39]. The drug Arbidol, active against influenza and HCV, targets the polar head of phospholipids in the membrane [40]. A class of rhodanine and thiobarbituric derivatives affects the fluidity of the lipid bilayer, thus compromising the efficiency of virus–cell fusion and preventing viral entry [41].

In conclusion, we characterized 17Y as a PBTZ broad-spectrum virucidal compound active against different enveloped viruses. Given the low toxicity for cells, the potential use of PBTZs also in disinfectants, repellents, skin creams, aerosol, sanitizing product and nasal spray could be of particular interest to prevent infections from different enveloped viruses, including flavivirus and coronavirus.

## Data Availability

Not applicable.

## Supplementary Materials

The following supporting information can be downloaded at: https://www.mdpi.com/article/10.3390/v14061157/s1. Figure S1. Virucidal activity of HeE15-2Y; Scheme S1. Synthesis of HeE15-2Y.
