# Supplementary material for: Virucidal Activity of the Pyridobenzothiazolone Derivative HeE1-17Y against Enveloped RNA Viruses
Source: Viruses. 2022 May 27;14(6):1157. doi: 10.3390/v14061157 (PMC9228864; doi:10.3390/v14061157)
Supplement: Supplementary file 1 [file viruses-14-01157-s001.zip › viruses-1701632-supplementary.pdf]

Supplementary material for the manuscript "Virucidal activity of the pyridobenzothiazolone derivative HeE1-17Y against enveloped RNA viruses" by Bonotto et al.,

HeE1-17Y **HeE15-2Y**

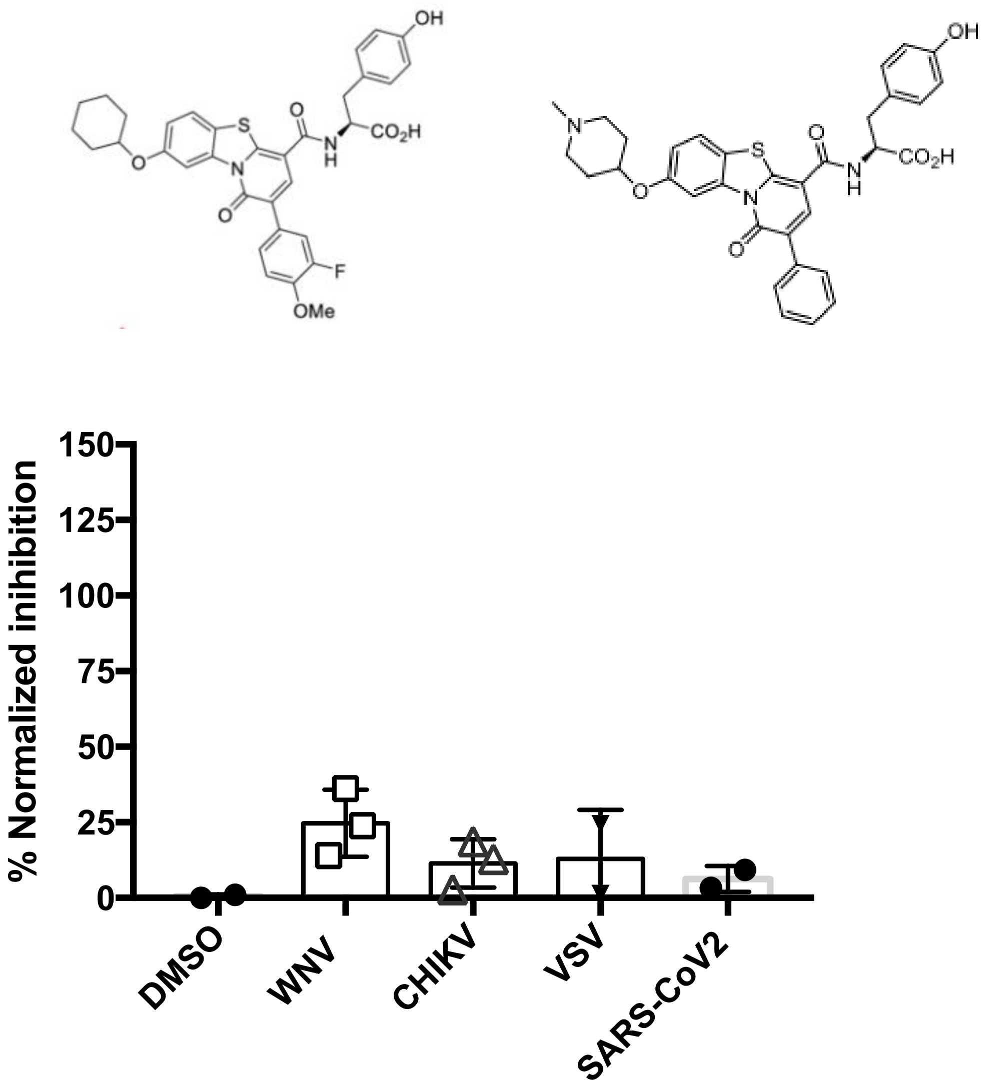

Figure S1. Virucidal activity of HeE15-2Y. **Upper panel:** Chemical structures of HeE1-17Y and HeE15-2Y; **Lower panel:** Virucidal activity of HeE15-2Y. HeE1-15-2Y was pre-incubated at the indicated concentrations with WNV (6 μM), CHIKV (6 μM), VSV (10 μM) and SARS-CoV-2 (6 μM) and processed as described in Figure 4.

General information for the synthesis of HeE15-2Y.

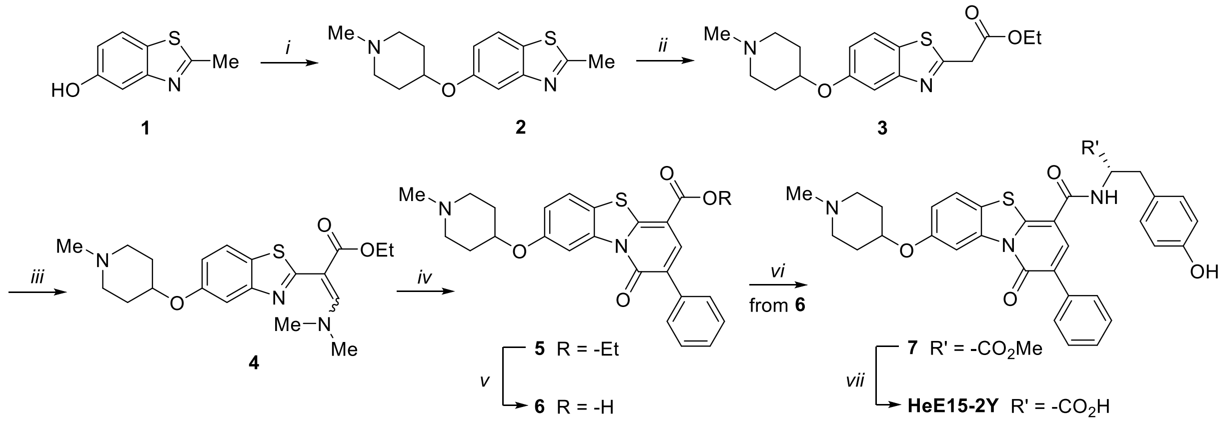

Scheme S1. Reagents and conditions: *i)* 1-methylpiperidin-4-ol, PPh_3, DIAD, dry THF, 0°C→rt, ultrasounds; *ii)* 60% NaH, diethylcarbonate, THF dry, reflux; *iii)* DMF-DMA, dry DMF, 80 °C; *iv)* phenylacetic anidride, neat conditions, 110 °C; *v)* 10% NaOH, MeOH, 70 °C; *vi)* L-tyrosine methyl ester hydrochloride, TBTU, DIPEA, dry DMSO, rt; *vii)* aq. 1N LiOH, 1,4-dioxane, rt.

All starting materials were commercially available, unless otherwise indicated. Reagents and solvents were purchased from common commercial suppliers and were used as such. Reactions under sonication were performed by an ultrasonic bath (VWR) with a frequency of 45 kHz. All reactions were routinely checked by thin-layer chromatography (TLC) on silica gel 60F254 (Merck) and visualized by using UV or iodine. Flash chromatography separations were carried out on Merck silica gel 60 (mesh 230-400), except for derivative **3** where aluminum oxide (activated, basic, Brockmann I, Sigma Aldrich) was used. Melting points were determined in capillary tubes (Büchi Electrotermal model 9100) and are uncorrected. Yields were of purified products and were not optimized. ^{1}HNMR spectra were recorded at 200 or 400 MHz (BrukerAvance DRX-200 or 400, respectively) while ^{13}CNMR spectra were recorded at 101 MHz (BrukerAvance DRX-400). Chemical shifts are given in ppm (δ) relative to TMS. Spectra were acquired at 298 K. Data processing was performed with standard Bruker software XwinNMR and the spectral data are consistent with the assigned structures. The purity of the compounds (≥95%) was revealed at 254 nm by HPLC analysis using a Jasco LC-4000 instrument equipped with a UV-Visible Diode Array Jasco MD-4015 (Jasco Corporation, Tokyo, Japan) and an XTerra MS C18 Column, 5 µm, 4.6 mm x 150 mm (Waters Corporation, Massachusetts, USA): flow rate, 0.5 mL/min; acquisition time, 10 min; gradient: acetonitrile/water (0 to 100% in 10 min).

Experimental part

2-Methyl-5-[(1-methylpiperidin-4-yl)oxy]-1,3-benzothiazole (2). To a stirred solution of starting 2-methyl-1,3-benzothiazol-5-ol **1** (2.70 g, 16.2 mmol) in dry THF (20 mL), 1-methylpiperidin-4-ol (2.81 g, 24.3 mmol), PPh_3 (6.43 g, 24.3 mmol) and DIAD (4.81 mL, 24.3 mmol) were added at 0 °C. Then, the mixture was stirred for 5 h at r.t. under sonication (ultrasound). After evaporation of the solvent under vacuum, the residue was poured into ice/water and extracted with EtOAc (x3). The combined organic layers were washed with brine (100 mL), dried over Na_2SO_4 and evaporated under vacuum to give a crude oil. After purification by flash column chromatography eluting with $CHCl_3$/MeOH 90:10, compound **2** was obtained as a yellow oil in 83% yield (3.54 g, 13.5 mmol). ^{1}HNMR (400 MHz, $CDCl_3$): δ 7.63 (d, J = 8.7 Hz, 1H, H-7), 7.43 (d, J = 2.4 Hz, 1H, H-4), 6.95 (dd, J = 2.4 and 8.7 Hz, 1H, H-6), 4.50-4.45 (m, 1H, OCH), 2.78 (s, 3H, NCH_3), 2.75-2.70 (m, 2H, piperidine-NCH_2), 2.45-2.40 (m, 5H, CH_3 and piperidine-NCH_2), 2.25-2.00 (m, 2H, piperidine-CH_2) and 1.75-1.60 (m, 2H, piperidine-CH_2).

Ethyl {5-[(1-methylpiperidin-4-yl)oxy]-1,3-benzothiazol-2-yl}acetate (3). Under N_2 atmosphere, to a suspension of 60% NaH in mineral oil (5.04 g, 126.0 mmol) in dry THF (30 mL), a solution of derivative **2** (3.30 g, 12.6 mmol) in dry THF (25.0 mL) was added dropwise. After stirring at r.t. for 20 min., diethyl carbonate (3.02 mL, 25.2 mmol) was added, and the reaction mixture was stirred at reflux for 11 h. Subsequently, the mixture was cooled at 0 °C with an external ice bath and quenched with EtOAc (10 mL) and then, water (5 mL), concentrated under vacuum and poured into ice/water. The aqueous phase was neutralized (pH = 7) adding 2N HCl, extracted with EtOAc (x3), and the combined organic layers were washed with brine (100 mL), dried over Na_2SO_4, and evaporated under vacuum to give a crude oil. After purification by flash column chromatography over aluminum oxide eluting with CH_2Cl_2/MeOH 99:1, compound **3** was obtained as an orange oil in 76% yield (3.18 g, 9.5 mmol). ^{1}HNMR (200 MHz, $CDCl_3$): δ 7.65 (d, J = 8.7 Hz, 1H, H-7), 7.45 (d, J = 2.4 Hz, 1H, H-4), 6.95 (dd, J = 2.4 and 8.7 Hz, 1H, H-6), 4.40-4.25 (m, 1H, OCH), 4.20 (q, J = 7.0 Hz, 2H, O$CH_2$$CH_3$), 4.10 (s, 2H, CH_2), 2.70-2.60 (m, 2H, piperidine-NCH_2), 2.40-2.20 (m, 5H, piperidine-NCH_2 and NCH_3), 2.00-1.70 (m, 4H, piperidine-CH_2 x2), 1.25 (t, J = 7.0 Hz, 3H, $OCH_2$$CH_3$).

Ethyl (2*E/Z*)-3-(dimethylamino)-2-{5-[(1-methylpiperidin-4-yl)oxy]-1,3-benzothiazol-2-yl}acrylate (4). Under N_2 atmosphere, to a solution of derivative **3** (3.00 g, 8.97 mmol) in dry DMF (6 mL), DMF-DMA (3.57 mL, 26.9 mmol) was added, and the reaction mixture was heated at 80 °C for 2 h. After cooling, the insoluble precipitate was removed by filtration and the filtrate was poured into ice/water and 10% NaOH in aqueous solution was added up to pH = 9. The mixture was extracted with EtOAc (x3) and the combined organic layers were washed with brine (100 mL), dried over

Na_2SO_4, and evaporated under vacuum to give a crude oil. After purification by flash column chromatography eluting with $CHCl_3$/MeOH 80:20, compound **4** was obtained as a yellow oil in 58% yield (2.03 g, 5.2 mmol). ^{1}HNMR (400 MHz, $CDCl_3$): δ 7.80 (s, 1H, CH), 7.65 (d, *J* = 8.7 Hz, 1H, H-7), 7.45 (d, *J* = 2.4 Hz, 1H, H-4), 6.95 (dd, *J* = 2.4 and 8.7 Hz, 1H, H-6), 4.45-4.35 (m, 1H, OCH), 4.15 (q, *J* = 7.0 Hz, 2H, OCH_2CH_3), 2.95 (s, 6H, $N(CH_3)_2$), 2.75-2.65 (m, 2H, piperidine-NCH_2), 2.40-2.25 (m, 5H, piperidine-NCH_2 and NCH_3), 2.10-2.00 (m, 2H, piperidine-CH_2), 1.85-1.70 (m, 2H, piperidine-CH_2), 1.35-1.20 (t, *J* = 7.0 Hz, 3H, OCH_2CH_3).

Ethyl 8-[(1-methylpiperidin-4-yl)oxy]-1-oxo-2-phenyl-1*H*-pyrido[2,1-*b*][1,3]benzothiazole-4-carboxylate (5). A mixture of derivative **4** (1.80 g, 4.62 mmol) and phenylacetic anhydride (2.35 g, 9.24 mmol) was heated at 110 °C for 2 h. After cooling, the residue was purified by flash column chromatography eluting with $CHCl_3$/MeOH 90:10, affording compound **5** as a yellow oil in 77% yield (1.65 g, 3.6 mmol). ^{1}HNMR (400 MHz, DMSO-d_6): δ 8.80 (d, *J* = 2.3 Hz, 1H, H-9), 8.20 (s, 1H, H-3), 7.80 (d, *J* = 8.7 Hz, 1H, H-6), 7.65-7.55 (m, 2H, H-2' and H-6'), 7.45-7.40 (m, 2H, H-3' and H-5'), 7.35-7.30 (m, 1H, H-4'), 7.10 (dd, *J* = 2.4 and 8.7 Hz, 1H, H-7), 4.45-4.35 (m, 1H, OCH), 4.27 (q, *J* = 7.0 Hz, 2H, OCH_2CH_3), 2.75-2.65 (m, 2H, piperidine-NCH_2), 2.40-2.20 (m, 5H, piperidine-NCH_2 and NCH_3), 1.95-1.80 (m, 2H, piperidine-CH_2), 1.65-1.55 (m, 2H, piperidine-CH_2), 1.30 (t, *J* = 7.0 Hz, 3H, OCH_2CH_3).

8-[(1-Methylpiperidin-4-yl)oxy]-1-oxo-2-phenyl-1*H*-pyrido[2,1-*b*][1,3]benzothiazole-4-carboxylic acid (6). A mixture of derivative **5** (1.50 g, 3.24 mmol) in aqueous 10% NaOH/MeOH (1:4) (45 mL) was heated at 75 °C for 2 h. After cooling, the mixture was concentrated, poured into ice/water, and acidified with 2N HCl up to pH = 7. The obtained precipitated was collected by filtration to give compound **6** as a pure yellow solid in 67% yield (0.94 g, 2.2 mmol); mp = 250-252 °C. ^{1}HNMR (400 MHz, DMSO-d$_6$): δ 9.00 (brs, 1H, H-9), 8.30 (s, 1H, H-3), 7.80 (m, 1H, H-6), 7.65-7.55 (m, 2H, H-2' and H-6'), 7.45-7.10 (m, 4H, H-7, H-3', H-4' and H-5'), 4.45-4.35 (m, 1H, OCH), 2.75-2.65 (m, 2H, piperidine-NCH_2), 2.40-2.20 (m, 5H, piperidine-$NCH_{2,}$ and NCH_3), 1.95- 1.75 55 (m, 2H, piperidine-CH_2), 1.65-1.55 (m, 2H, piperidine-CH_2).

Methyl *N*-({8-[(1-methylpiperidin-4-yl)oxy]-1-oxo-2-phenyl-1*H*-pyrido[2,1-*b*][1,3]benzothiazol-4-yl}carbonyl)-L-tyrosinate (7). Under N_2 atmosphere, to a mixture of derivative **6** (0.90 g, 2.07 mmol) and L-tyrosine methyl ester hydrochloride (0.62 g, 2.69 mmol) in dry DMSO (15 mL), TBTU (0.86 g, 2.69 mmol), and DIPEA (1.62 mL, 9.32 mmol) were added. After stirring at r.t. for 2 h, the reaction mixture was poured into ice/water, acidified with 2N HCl up to pH = 6 and extracted with EtOAc (x3). The combined organic layers were washed brine (100 mL), dried over Na_2SO_4 and evaporated

to dryness to give a pure yellow solid in 59% yield (0.75 g, 1.2 mmol); mp = 291-292 °C. ^{1}HNMR (400 MHz, DMSO-d_6): δ 9.25 (s, 1H, OH), 9.00 (d, J = 7.8 Hz, 1H, NH), 8.90 (brs, 1H, H-9), 8.45 (s, 1H, H-3), 7.80 (d, J = 8.7 Hz, 1H, H-6), 7.75-7.65 (m, 2H, H-2' and H-6'), 7.45-7.40 (m, 2H, H-3' and H-5'), 7.35-7.30 (m, 1H, H-4'), 7.10 (d, J = 8.7 Hz, 1H, H-7), 7.10-7.00 (m, 2H, H-2'' and H6''), 6.60-6.50 (m, 2H, H-3'' and H-5''), 4.50-4.45 (m, 1H, CH), 4.40-4.35 (m, 1H, OCH), 3.65 (s, 3H, OCH_3), 3.10-3.00 (m, 2H, CH_2), 2.75-2.65 (m, 2H, piperidine-NCH_2), 2.40-2.20 (m, 5H, piperidine-NCH_2 and NCH_3), 1.95- 1.75 (m, 2H, piperidine-CH_2), 1.65-1.55 (m, 2H, piperidine-CH_2).

***N*-({8-[(1-methylpiperidin-4-yl)oxy]-1-oxo-2-phenyl-1*H*-pyrido[2,1-*b*][1,3]benzothiazol-4-yl}carbonyl)-L-tyrosine (HeE15-2Y).** A solution of derivative **7** (0.60 g, 0.98 mmol) and aqueous 1N LiOH (4.95 mL, 4.90 mmol) in 1,4-dioxane (8 mL) was stirred from 1 h at r.t. Then, the reaction mixture was poured into ice/water, acidified up to pH = 7 with 2N HCl to give a precipitate which was filtered. After crystallization by *n*BuOH, compound **HeE15-2Y** was obtained as a yellow solid in 34% yield (0.20 g, 0.33 mmol); mp = 219-221 °C. ^{1}HNMR (400 MHz, DMSO-d_6): δ 9.25 (s, 1H, OH), 8.95-8.75 (m, 2H, NH and H-9), 8.45 (s, 1H, H-3), 7.80 (d, J = 8.7 Hz, 1H, H-6), 7.75-7.65 (m, 2H, H-2' and H-6'), 7.45-7.40 (m, 2H, H-3' and H-5'), 7.35-7.30 (m, 1H, H-4'), 7.20 (dd, J = 2.4 and 8.7 Hz, 1H, H-7), 7.10-7.00 (m, 2H, H-2'' and H-6''), 6.60-6.50 (m, 2H, H-3'' and H-5''), 4.60-4.55 (m, 1H, CH), 4.50-4.45 (m, 1H, OCH), 3.10-3.00 (m, 1H, Tyr-CH_AH_B), 2.90-2.80 (m, Tyr-CH_AH_B), 2.75-2.65 (m, 2H, piperidine-NCH_2), 2.35-2.25 (m, 2H, piperidine-NCH_2), 2.20 (s, 3H, NCH_3), 2.10-2.00 (m, 2H, piperidine-CH_2), 1.95-1.85 (m, 2H, piperidine-CH_2); ^{13}CNMR (101 MHz, DMSO-d_6): δ 174.07, 164.25, 161.24, 156.14, 155.90, 151.60, 138.86, 136.71, 133.87, 130.33, 129.31, 128.80, 128.37, 127.72, 123.06, 122.33, 121.27, 116.37, 115.34, 107.65, 105.58, 72.53, 55.51, 52.09, 45.20, 36.31, 30.19. HPLC retention time (4.18 min).
